# Supplementary material for: HIVprotI: an integrated web based platform for prediction and design of HIV proteins inhibitors
Source: J Cheminform. 2018 Mar 9;10:12. doi: 10.1186/s13321-018-0266-y (PMC5845081; doi:10.1186/s13321-018-0266-y)
Supplement: Supplementary file 2 — Additional file 2. Source code of HIVProtI web server. [file 13321_2018_266_MOESM2_ESM.zip › HIVprotI_Source-code/hivprotI/jsme/api_javadoc/export/client/package-summary.html]

export.client


JavaScript is disabled on your browser.


Skip navigation links


- Package
- Class
- Tree
- Deprecated
- Index
- Help

- Prev Package
- Next Package

- Frames
- No Frames

- All Classes

# Package export.client

- Class Summary

  | Class | Description |
  | --- | --- |
  |  |  |
  | --- | --- |
  | JSME |  |
  | Utils |  |

Skip navigation links


- Package
- Class
- Tree
- Deprecated
- Index
- Help

- Prev Package
- Next Package

- Frames
- No Frames

- All Classes
